# The design of the “autistics in (educational) space: building our own futures” doctoral project

**DOI:** 10.1177/13623613251328495

**Published:** 2025-03-29

**Authors:** Ryan B Collis

**Affiliations:** York University, Canada

**Keywords:** autistic, education, high school, science fiction

## Abstract

As an autistic researcher and doctoral candidate, I have designed my dissertation research in a way that values the lived experience of my four autistic participants. Using their responses to a series of material objects and a science fiction novel by, and about, an autistic person, I hope to find new and innovative ways to reconceptualize inclusive education in high school. This article explains my theoretical framework and methodology, as well as some preliminary results and discussion.

**Lay Abstract:**

I am autistic and a PhD student and I look for ways to learn from other autistic people. I gave a group of 4 autistic participants 14 items and asked them to do something with each of them, then send me pictures of what they did. We also all read a science fiction novel written by an autistic author and talked about what we thought was interesting or that felt familiar to us. By using what they shared with me, I want to find ways to make high school more comfortable for autistic students. In this article, I describe how I came up with this plan, what I did, and some of the first things I discovered.

## Introduction

The increase in the number of Disabled students^1^ in non-segregated classes is a mark of the success of the education system. When students are moved from a segregated system to one where they are provided support in classes with their peers, we can expect improvements in their academic achievement, sense of social belonging, and their engagement in school (Barron et al., 2023). In order to learn how to best serve these students, we need new approaches to research what autistic people actually need—ones that are respectful, inclusive, and participant-led because often research on ways to help Disabled people produces solutions that Disabled people themselves do not want, welcome, or use (Maaß & Buchmüller, 2018).

My doctoral research seeks to find new ways to support autistic high school students by talking to autistic people with lived experience (Hamraie, 2019; Milton, 2014; Woods & Waltz, 2019) of how well, or poorly, the school system supported them. In particular, I seek to examine what Dokumaci (2018) called the “micro-activist affordances” that participants create when confronted by “environmental affordances” that were not designed for them. As an autistic person, I can bypass some of the difficulties that Heasman and Gillespie (2019) discuss regarding ethnographic research that relies on “autistic-to-neurotypical interactions which take place against the cultural backdrop of neurotypical norms and expectations” (p. 910). Heasman and Gillespie (2019) attribute this difficulty, what Milton (2012) termed the “double empathy” problem, to autistic communication lacking a social protocol. While my autistic communication style provides me some advantages, it is still necessary to find a way to conduct research where I neither look like a doctor making a diagnosis and prescribing a cure, nor cast myself in the role of a servant by letting the research participants set the entire direction of the study (Gaver et al., 1999).

My research avoids the above pitfalls using two methodologies: *Cultural Probes* and *Shared Reading*. Cultural probes are a way to gain insight into the actual needs of research participants, allowing the investigator to lead a discussion without dominating it (Gaver et al., 1999). While cultural probes have been used in design research for decades, they have not been widely used in education or disability research. Cultural probes are a collection of physical items to interact with or tasks to accomplish that are provided to the participants. Through analyzing the results of their interactions, an impression of their lives and experiences can be gleamed. In addition, to avoid relying on binary, neuronormative ideologies, this research design used *Shared Reading*, a “creative and open methodology[y] that provide an immersive shared experience” (Chapple, Davis, et al., 2021, p. 3). This method involves both the participant and the researcher reading a text divided into sections and meeting multiple times to discuss their reactions to it.

In this article, I will describe how I use cultural probes and shared reading in my research on making inclusive classrooms that better serve autistic students. By using a selection of different cultural probes with autistic adults who have been through the public education system, I will learn from them what did or did not help them be successful so that barriers can be eliminated, and success can be replicated. I will then describe my shared reading ethnographic method, which uses the independent, but co-operative, reading of a text as the starting point for collaborative conversations.

## Positionality

I am a white, cis-gendered, heterosexual autistic male. Because the learning disabilities associated with my autism (including dyslexia and disgraphia) were identified in my third year of university by a concerned and understanding Computer Science professor who recommended I access Student Accessibility Services I was able to receive accommodations. Those accommodations allowed me to complete my degrees. Not all students are as fortunate. I believe it is important that I use the opportunities that my education provides to ensure that other students also have the same chances to succeed in their education that I did.

## Literature review

### Autistic students and inclusive education

Information on the lived experience of autistic high school students is limited, though there has been some research on this topic. Zakai-Mashiach (2025) notes that most of this research focused on the perspectives of other “stakeholders” such as parents and teachers, with a smaller number of first-person studies. They noted that these studies usually looked at sections of the educational journey, rarely the entire 12 (or more)-year-long continuum. They argue that the research on autistic in “general school settings” (as opposed to segregated schools) “has revealed mainly negative experiences and has indicated that most autistic children are at risk of negative encounters such as bullying, anxiety, social isolation and loneliness” (p. 2). Lebenhagen (2024) notes that there are multiple reasons why attempts at inclusive education fail: inadequate teacher training, medical model influences on government language, and neo-liberal trends toward individuality. They also argue that inclusive education discourse is complicated by “assertions from critical disability theorists that while the objective of integration may strive to create a more inclusive society, integration is a modern form of oppression because it aims to purify and normalize through assimilation” (p. 14). Zakai-Mashiach (2025) would appear to agree, noting that “No studies have been found that describe the lived experiences of autistic students in general schools as positive and empowering” (p. 2). At issue here is the difference between integration and inclusion. As my colleagues and I wrote in Barron et al. (2024), “inclusive education extends beyond mere integration to include an emphasis on creating a culture of inclusion, de-stigmatizing disability, as well as valuing and presuming the competence of all students” (p. 49). What we called “mere integration” is what Lebenhagen (2024) is referring to above—putting autistic students in regular classes without the necessary accommodations and supports. Naraian (2011, 2020, 2021) examines how inclusion can be created without falling into assimilation, where the included student is expected to take on the role of a non-disabled student. Naraian (2011) argues that teachers who are thoughtful and deliberate in their efforts “contribute significantly to making nonnormative behaviors and actions accessible” to their class to help disabled students bond with their peers. “This, in turn, can stimulate the creation of alternative norms—an activity that lies at the heart of inclusive efforts” (p. 259). Naraian (2021) assumes that “inclusive” education must lie somewhere other than within the divided school tracks and scholarship of “general education” and “special education” while “the practice of ‘inclusive’ education may inevitably encompass both ‘general’ and ‘special’ education” (p. 299). However, as Ashby (2012) reminds us, “The separation between general and special education is neither natural nor inevitable” (p. 98). They encourage the use of disability studies to engage in critical thinking about what education for all students can be and a way to reorient the goal of special education from “remediating supposed defects” to educational success (p. 96). Their argument, that disability studies is not a replacement for special education, but a way to rethink what education means, also requires a change in who is defined as an expert. They write that the way most of the research on disability is done centers “so-called scholars and experts on disability” while marginalizing disabled people, and fails to “learn from the individual, not about the individual” (p. 95).

### Speculative research using speculative fiction

Tuttle (2005) reminds us that the political structures that shape our world do not magically appear fully formed, nor are they infinitely enduring. They are the result of one event leading to the next, and that “some connections are more logical and likely than others” (p. 39). Speculative methods of research using science fiction allow for investigations of these connections by making the world strange. Suvin (1979) uses the term “cognitive estrangement” to describe “the way science fiction estranges or distances readers from their knowledge and assumptions about what constitutes reality in order to move them to question those very assumptions” (Schalk, 2018, p. 114). Schalk (2018) uses the term “defamiliarization” “to refer to the way speculative fiction texts make the familiar social concepts of (dis)ability, race, gender, and sexuality unfamiliar in order to encourage readers to question the meanings and boundaries of these categories” (p. 114). Slater (2024) argues that the critical function of cognitive estrangement is to make clear that reality is a social construction, that history is mutable, and to provide the opportunity to imagine alternative futures.

Speculative fiction allows for addressing issues of race even without the presence of racialized humans by mapping racial identities on aliens and other non-realist beings (Schalk, 2015). Likewise, through speculative fiction’s focus on “changing bodyminds, technology (medical and otherwise), and new species is very much about the concepts of disability and ability” (Schalk, 2015, p. 23). Schalk (2015) uses the feminist disability studies term “bodymind” to refer “to the inextricability of mind and body which seeks to emphasize how processes within and outside of our being impact one another in a myriad of unpredictable ways that cannot be cleanly divided from one another” (p. 22). In later writings, Shalk (2018) describes disability in black women’s speculative fiction as “complex embodiment” that requires acknowledgment of the “positive, negative, and ambivalent aspects of disability (physically, mentally, and socially) as well as the relationship between all three” (p. 24). Disability representation in science fiction can take multiples forms. The disabilities can be real disabilities projected on future peoples, or fictional ones created by the authors for a specific purpose while the disabled characters can either be real or imaginary beings. Schalk (2018) describes the use of defamiliarized realist disabilities (disabilities that exist in the real present) being applied to “the nonrealist bodyminds of demons, werewolves, and half-mortals” (p. 118) to “make these realist disabilities less clearly knowable or predictable than expected” (p. 119). This encourages readers to think about disability from the character’s perspective, not from their preconceptions of disability. Likewise, the use of nonrealist disabilities means the reader has no preconceptions and must rely on the text (Schalk, 2018). Both approaches challenge “readers’ assumptions about the meanings, manifestations, and effects of a particular disability on physical, mental, social, and environmental levels alike, forcing readers to reconsider what they know or think they know about what it means to be disabled” (Schalk, 2018, p. 119). Slater (2024) argues that because much of what is familiar is the product of reification and pacification through normalization, this defamiliarization of oppression develops “critical consciousness and the capacity for historicized thought” (p. 71). The ability for speculative fiction to provide unlimited possibilities to resist normalization “have important liberatory potential for marginalized people” (Schalk, 2015).

Slater (2024) describes “sociological imagination” as the ability to connect one’s personal experiences with the “socio-historical forces that produce them” (p. xi). Koro et al. (2024) claims that the “social imagination” provides the ability “to envision what should be and might be” while it is the “radical imagination” that provides the “capacity to think critically, reflectively, and in innovative ways about social worlds” (p. 677). Koro et al. (2024) examines how speculative approaches (to research, philosophy, and scenario building) can inform qualitative inquiry. They suggest a “prefigurative methodology” that borrows from a desired future can enable scholarship that approaches the future as a place of possibilities and a “space for radical transformation” (p. 677). They argue that this is not invention from nowhere because it begins with “a point of departure that enables a coherent and rigorous speculative process of thought” (p. 679). In science fiction, this departure is called the novum, the element of the story that differs from reality and creates the cognitive estrangement that distances readers from the familiar (Slater, 2024). Koro et al. (2024) claim that methodologies must build not just on possibilities but also on the impossible and the speculative, as such scholarship can “move social sciences toward imaginative futures, futures which move beyond individual subject’s histories and presence while being shaped by the subject’s past” (p. 688). They argue that this will allow for the disruption of institutionally static and conventional ideas of the future and the imagining of “different, more equitable and responsible educational future subjects” (p. 688).

### Disability studies as method

Minich (2016) points out that there exists deficit-focused research on disability that would not be considered to belong to the field of disability studies, while other research done by disabled scholars and activists that disability scholars do not see as critical disability studies. They suggest a new approach to disability studies where the focus is on mode of analysis instead of objects of study. This reconfiguring, they argue, makes clear that research on disability is not automatically disability studies, and research that does not look directly at disability can still be disability studies. Minich (2016) describes this “methodology of disability studies” as a means of focusing on social norms that make particular attributes become impairments and how stigma is attached to specific attributes in particular populations. This methodology is intentional, Minch argues, and works “with the goal of producing knowledge in support of justice for people with stigmatized bodies and minds” (p. 3). Minich (2017) uses Schalk’s “distinction between (dis)ability (‘a system of social norms which categorizes, ranks, and values bodyminds’) and disability (‘a historically and culturally variable category within this larger system’)” (p. 1) as an important way to study “power, privilege, and oppression of bodily and mental norms which is not dependent upon the presence of disabled people, yet is informed by social perspectives, practices, and concerns about disability” (Schalk, 2017, p. 3). This approach also allows for disability to include any non-normative existence, regardless of current definitions (Schalk, 2017).

Schalk (2015) points out that black speculative fiction, especially the genre know as Afrofuturism, is known for imaging ways the distribution of medical technologies and advancements will favor wealthy white populations over others. Thus, scholars of disability and race must identify how ablism, racism, sexism, and classism shape (dis)ability. It is because of these kinds of connections between disability and fiction that Schalk (2015, 2017) suggests that disability studies can be understood as a method for analyzing (dis)ability in speculative fiction, a method that can examine “how disability is defined and redefined in non-realist worlds where the expectations, possibilities, and limits of bodyminds are different” (2015, p. 24). Schalk (2017) follows Minch in understanding critical disability studies can be “a method, an approach, a theoretical framework and perspective—not (exclusively) a study of disabled people” (p. 2). Schalk (2015) also argues that scholars using disability studies as method to analyze speculative fiction must be able to demonstrate that a character is or is not disabled in the context of the text. This use of disability studies as method must also consider how technology, especially technology that interacts with disability, is presented, because “technology is neither benign nor objective, but rather is created and used within particular social and historical contexts of privilege and oppression” (Schalk, 2018, p. 104). Schalk (2018) argues that a future without disability thanks to technology may sound positive, but questions must be asked: Who benefits from, or has access to, this technology? Whose bodyminds will the technology be tested on? Whose labor will provide the materials for this technology? Schalk (2018) reminds us that historically “people of color, women, working-class people, and people in poverty will benefit the least from technological advances and will be most at risk for harm in the development, production, and consumption of new technologies” (p. 106).

### Research questions

It is necessary to begin this research by unsettling the status quo around “knowing the classroom” and rendering the invisible visible as described by Dokumaci’s (2018) description of the “habitus of ableism.” To do this, I will employ cultural probes (Gaver et al., 1999) to learn in an oblique way how my participants remember getting through their time in school. This also seeks to examine how expertise in education has been constructed by considering Dokumaci’s (2018) “sophisticated lay scientist” and Collins’ (2014) “experience-based experts” to interrogate how [certifying/permitting/endorsing/qualifying] certain knowers disrupt the construction of the “expert.” The second strand of research uses a science fiction text as a shared experience between the researcher and the participant to aid in reflections on living an autistic life. Often the micro-affordances a disabled person makes to move through the world fade into habit and are forgotten. By reading a science fictional account of an autistic person in the far future, some of these techniques will become visible. Tomin (2021b) has her participants use “science fictional storytelling techniques” to “envision radically different educational futures” because SF “allows us to imagine future possibility and societal change” (p. 242). She argues that SF can be mobilized as subversive literature “through which we can imagine otherwise; to build upon the past, critically interrogate the present and write in pursuit of possible futures” (p. 242).

For this research project, there are two research questions:

1. What can we learn about the experiences of autistic students through indirect study methods that can improve the education system?
2. How can we use science fictional accounts to render the invisible visible, in line with Dokumaci’s (2018) “habitus of ableism”?

## Theoretical framework

Schalk (2017), following Minich (2016), understands critical disability studies as many things at once—method, approach, theoretical framework, and perspective. Minich (2016) asks how a scholar can claim to be “doing disability studies” if they are not considering “how attendance policies, seating arrangements, assignments, lighting, and mode of instruction” affect the accessibility of the classroom environment (p. 4). In her paper “Disability as Method: Interventions in the Habitus of Ableism through Media-Creation,” Dokumaci (2018) likewise used disability as a means of theorizing about accessibility, describing the micro-activist affordances disabled people employ to move through spaces that were created without expecting their non-normative bodyminds. Here, bodyminds is used in the way Schalk (2018) described it, reliant on the “inextricability of mind and body” (p. 5). Through what Dokumaci (2018) calls “the habitus of ableism,” these micro-activist affordances are hidden from those for whom the presence of unexpected bodyminds is improbable or unthinkable. At the same time, these non-normative bodyminds risk stigmatization if their affordances are detected. Goffman (1986) asks, “does the stigmatized individual assume his differentness is known about already or is evident on the spot, or does he assume it is neither known about by those present nor immediately perceivable by them?” (p. 13). If the individual can hide their micro-activist affordances and seem to fit into their environment, it may be possible to avoid the stigma of their disability, but in other cases the tools necessary to exist in that environment may be the source of the stigma.

## Methods

### Cultural probes

Cultural probes are a collection of objects that serve as a common language for both researchers and participants, expressing the research focus and providing a simple and interesting means of expression for the participants, while offering “a basis for joint learning, for negotiating interpretations, discussing perspectives and identifying problems” (Maaß & Buchmüller, 2018, p. 125). They are physical objects positioned for the participants to interact with, and through those interactions the researcher gains a unique view of the participant’s life.

A cultural probe is an item or task that the participant interacts with to produce a physical product^2^ that can be interpreted by the researcher to learn more about the participant. Thoring et al. (2013) fit these items into 10 categories:

Wrapping—packaging or container for the rest of the probes;
Diary—to collect participants’ structured or unstructured thoughts;
Maps—to mark participants’ memories or thoughts about a location;
Framework—an abstract structure for different purposes: time-related (e.g. Gantt charts), value-oriented (e.g. Venn diagrams), or abstract mappings (e.g. postcards);
Photo/video documentation—taken by the participants at researcher’s direction;
3D tools—physical objects the participants interact with;
Random probes—unstructured activities for the participants to engage in;
Surveys—to acquire qualitative data;
Gifts—items to thank and motivate participants;
Supporting materials—tools, prompts, and instructions for interacting with the probes.
(pp. 6–7).

Thoring et al. (2013) also identified the six possible functions a probe could serve:

Documentary—these probes capture participants’ observations and activities;
Visionary—these probes capture emotions and wishes;
Inspirational—these probes inspire the participants in a non-physical way;
Motivational—these probes keep participants engaged through a physical reward;
Practical—these probes serve a practical purpose (such as a pen for writing);
Instructive—these probes direct the participants in their use of the probes.
(p. 7).

**Figure 1.**
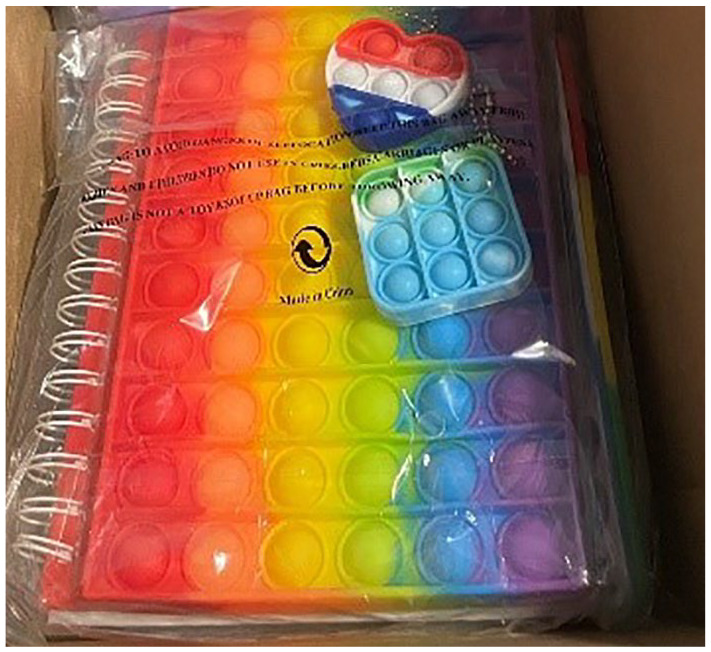
Fidget diaries.

Some probes might have multiple categories and/or functions, such as the fidget diaries (see Figure 1) I sent to my participants; they fit into both the diary and gift categories and were both documentary and practical.

In examining research that mobilized cultural probes, I found records of the diversity of items used in different projects, including maps, cameras, photo albums, and media diaries (Gaver et al., 1999); diaries, task books, and projective tools (pens, stickers, markers, etc.) (Celikoglu et al., 2017); and diaries, pairs of linen bags (one each marked with a smiley or sad face), cameras, and postcards (Maaß & Buchmüller, 2018). Cultural probe packages usually also include a list of tasks for the participant to do and the equipment necessary for them to carry out the tasks, such as documenting experiences using a camera, art supplies, or stickers. These packages need to encourage the participants to do the requested tasks while also making them feel that they are being treated as experts (what Collins (2014) calls experience-based experts).

**Figure 2.**
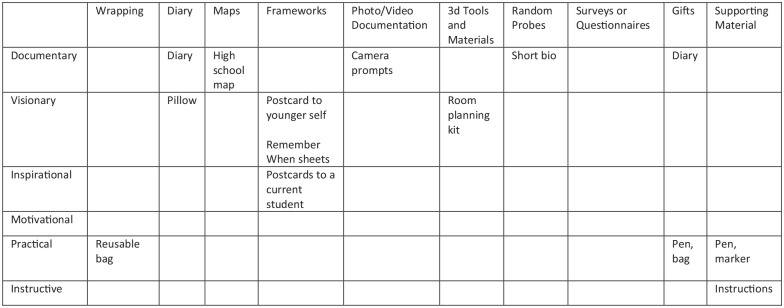
Probe matrix.

**Figure 3.**
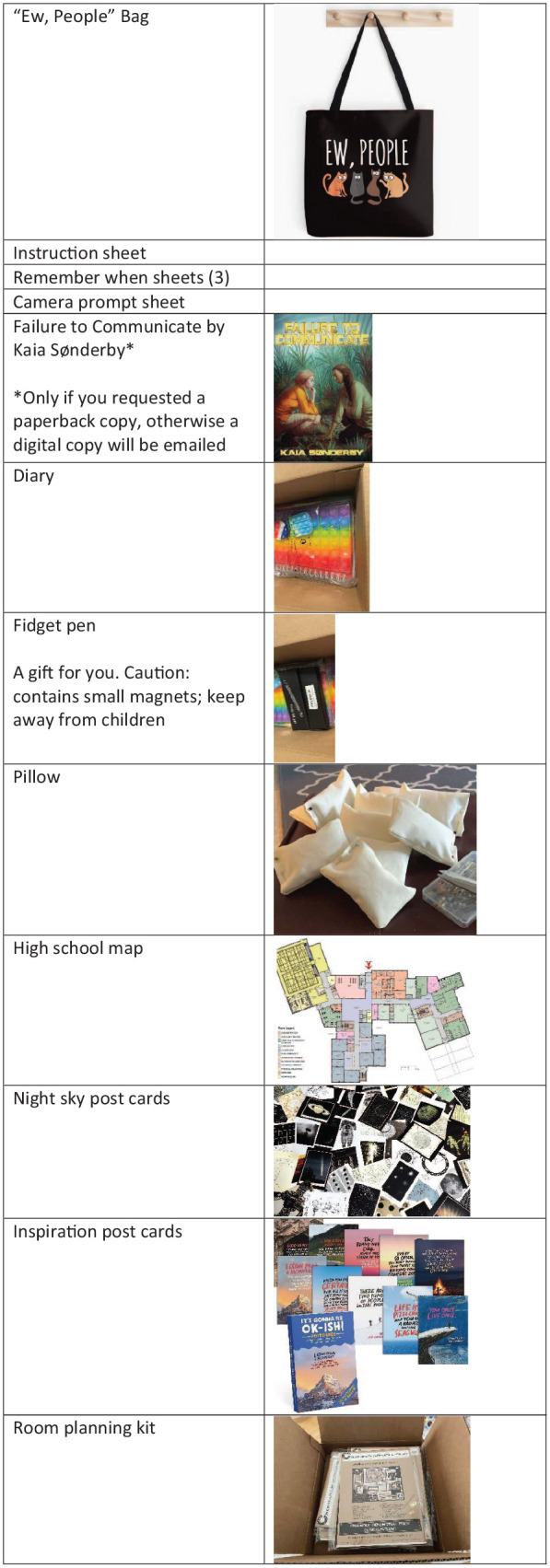
Probe package contents.

To establish my own protocol, I used Thoring et al.’s (2013) explanation of the different categories and functions of probes and created a 10 × 6 matrix, with the intention of having at least one entry in each of the 10 categories and two in each of the six functions (see Figure 2). Some of my selections include Wallace et al.’s (2022) pillow probe and Burrows et al.’s (2015) camera prompts and “Remember When” probes. By drawing on a variety of cultural probe categories and functions, this methodological approach will provide thick descriptions (Leeds-Hurwitz, 2015) of the lived experiences of my autistic participants. Not all possible categories or functions of probes were included, and some probes served multiple purposes (see Figure 3). For example, the “Ew, people” bag was a wrapping, a gift, and practical while the fidget pen was a practical gift and supporting material. In the instructions, I provided a possible timeline to complete the probes, but the participants were able to take them on in any order or time frame they wished.

Celikoglu et al. (2017), in considering a variety of methodologies, noted that ethnographies are useful because they provide contextual information on how people live while surveys—their epistemological opposite—“elicit answers to questions posed from the perspectives of producers or designers, leaving respondents no space to respond on their own terms” (p. 85). They found cultural probes struck a balance between “the impositions of structured surveys and the broader outcomes of ethnographies, eliciting design-relevant information while preserving the conceptions of users” (p. 85) because of their subjectivity, openness, and ability to provoke discussion. This method is sufficiently flexible to work in a wide range of research situations. For example, Paay et al. (2009) note that by 2009 this method had been successfully used to study care facilities for elderly people, design in a sensitive health care setting, intimacy in couples, and for data collection for a domestic design process. The results provided by cultural probes are impressionistic accounts of participants’ beliefs, desires, and cultural concerns (Gaver et al., 1999), providing a look into the participants’ history, present, and providing inspiration into future designs (Paay et al., 2009).

### Shared reading

A methodology based on shared reading could potentially be an effective way to avoid the binary ideologies that often impede autistic participation in research. Chapple, Davis, et al. (2021), following Longden et al. (2015), believed that discussing fiction promoted communal thinking and encouraged “explorations of individual differences” (p. 3). Longden et al. (2015), citing Dowrick et al. (2012), described shared reading as a “socially coalescing presence, allowing readers a sense of subjective and shared experience” (p. 1). In his book, *See It Feelingly* (2018), Savarese engages in a form of shared reading with well-known autistic partners. He describes this work as “intentionally, if casually, ethnographical” in that he converses with his “partners” and reports what they have to say about the novel and their experiences (p. 12). This is a newer way of engaging in ethnography, one done through a framework of dialogue that “displaced the politically charged, asymmetrical metaphor of ‘reading over the shoulders of natives’ with that of ‘reading alongside natives’” (Lassiter, 2005, p. 5).

My goal with this research is to make noticeable the micro-activist affordances that each participant was forced to create. These are often rendered invisible by what Dokumaci (2018) calls the “habitus of ableism.” I wanted to avoid a text where the character was presented as a “super crip” (Davis et al., 2018, p. 30) who only experienced positive aspects of autism. Rather, the story had to show a realistic and balanced portrayal of being autistic to give us something to collaboratively engage with.

I chose to read fiction, instead of non-fiction, because “fiction encourages an overcoming of social pressures and conformity” (Chapple et al., 2022, p. 3). Furthermore, in research, autistics have shown a preference for fiction from the science fiction and fantasy genres (Chapple, Williams, et al., 2021). SF, as a genre, is defined by the way it holds the present up for critique by extrapolating where we as a people might end up if we continue (or do not continue) on our current path. Other scholars have written about how science fiction is “deeply ingrained in contemporary social consciousness as a way to process change” (Tomin, 2021a, p. 3). Educational researchers have shown that studying science fiction is a way to shine a spotlight on how things are now by imagining how they could be different, and this can engage learners through the “excitement of identifying and describing social forces at work in their world” (Passell, 2013, p. 61). Furthermore, Savarese (2018) notes that autism and science fiction go together like “two astronaut peas in a spaceship pod” (p. 89), and so reading science fiction by an autistic author, about an autistic protagonist, with an autistic research partner, appeared to be an excellent way to learn about their experiences and to mobilize science fiction as “a subversive literature through which we can imagine otherwise; to build upon the past, critically interrogate the present and write in pursuit of possible futures” (Tomin, 2021b, p. 242).

Prior to each online meeting, the participants and I read a section of the novel (roughly 50 pages or 1/6th) and made notes on anything that stood out as something we wanted to share. While the participants were not given instructions on how to determine what met the criteria for being “shareworthy,” I used a more methodological approach to code the text as I went, noting passages that fell into one of six categories: communication, sensory, accommodations, mental/inner voice, social/reading people, and neurodivergent thoughts or behaviors. I then made a list of 5–10 of what I considered the most important, relevant, or significant of these passages to share at the meeting with the participants. In each meeting, I had the participant share their observations on the novel section first and then raised any points I had flagged that they had not. This ensured that there would always be at least 5–10 taking points per session even if the participant did not have their own observations to share.

For each of the four participants there were six meetings, with three of them having meetings on average every 2 weeks and one meeting weekly. There was some variance to this schedule when a participant became unavailable and needed to reschedule. Meetings were scheduled to take 1 h, although the first meetings were much shorter. The recordings (nearly 24 h worth) were sent for transcription, and the transcripts were coded thematically using the software QualCoder 3.3.

### Community involvement statement

The research question and study design were developed by the author, an autistic doctoral student, and high school teacher. Modifications to the original protocols were made by the participants, all of whom are autistic. This project is being undertaken under the precepts of Community-Based Participatory Research (Bidwell, 2009; Nicolaidis et al., 2019), so as a component of the full dissertation the participants will decide how to disseminate the results with the author providing support. Participant anonymity is a requirement of the ethics certificate, so they will not be named in this article.

## Results

For recruitment, following ethics approval (York University Human Participants Review Committee certificate number STU 2022-097), I contacted the Student Accessibility Services (or equivalent) at all 23 universities in the province of Ontario. Only one sent my call for participants to their autistic students. As a result, three of my four participants are from one university. My final participant was recruited by word of mouth. After signing a written informed consent form provided in PDF and Word format, the participants were sent a set of cultural probes and a physical copy of the book *Failure to Communicate* (Sønderby, 2017), a novel about the only autistic human in the galaxy who serves on a spaceship as an expert in understanding new forms of sentient life, despite her difficulties in understanding her own. My participants were

White male, age 29, diagnosed first year of university;
Indigenous male, age 20, non-speaking, age of diagnosis unknown;
White female, age 20, diagnosed age 8 or 9;
White female, age 28, diagnosed age 21.

Data on socioeconomic status were not collected.

The participants submitted photos of their work with the probes over a several month-long period. In comparing the responses to probes across participants, I found some patterns. When asked to write a postcard to their younger self, three of the four participants comforted themselves with messages that life would be better in the/their future. This included phrases such as “weather[ing] the storm,” “things will get better soon,” and “you have survived 100% of worst days.” These all seemed to relate to the difficulties they experienced in high school, with one participant directly stating that “highschool was hell but it gets so much better.”

Another common thread was three of the four felt it important to tell their past self that they would form and/or repair relationships. One wanted to tell herself that she has “an amazing boyfriend you just celebrated 4 years with” and to “remember you are loved.” Another included a postscript in their letter saying they “get the best dog & family ever one day!!!,” while the third explains to her younger self that “you aren’t broken, you’re asexual and aromantic” and she will one day have “people in your life that you’ll love in other ways and they’ll love you back the same,” as well as that “one day, Mom and Dad will take you seriously.”

With regard to the shared reading, all participants found the autistic character to be very relatable, and her struggles mirrored their own experiences. One participant, who usually doesn’t like reading fiction, noted, “But, like, this book I found I’ve enjoyed it a little bit more because I find myself identifying with some things the character says about how they feel in certain situations.” Another participant found the main character’s, Xandi, mental struggles “very reliable [sic]. I yell and scream and get a bad reaction from my brain and chest and tic a lot.”

Following Chapple, Davis, et al. (2021), I use the novel as “common ground” that provides “a shared social setting to operate within during discussion sessions: hence discussion was not just ‘about’ but ‘around’ and ‘within’ the book” (p. 7). Thus, I was able to draw on the participants’ experiences with the education system through discussion that targeted the ways participants did or did not relate to the text. One participant, reflecting on how his learning changed after leaving high school, noted “I love math now and that was a late in life kind of discovery because the education system kind of let me down.” He elaborated that he

didn’t find out I was a fucking math savant until I was twenty-seven. In fact, your education system suppressed that in me, because it didn’t allow me to function how I needed to function to be focused on my work, my school work. How can I—how can you expect me to learn about math when I’m like, fucking, just trying not to let everyone find out I’m kind of gay, you know?

Another participant agreed that the post-secondary system was a better fit for them: “I have experienced social struggles and have to advocate for my own accommodation and have thrive [sic] in a campus setting compared to the K-12 setting.” They found that the K–12 system prevented them from accepting and appreciating their differences: “In elementary and high school I had thoughts where I wished I was like a neurotypical person” in part because “I often hit myself and self harmed and was a perfectionist in high school.”

One theme that came up was that the participants felt that teachers were not prepared to work with autistic students. One participant stated that her grade four teacher

was like, “Why are you doing—why are you different? Why is this not working?” And I think it was less malicious of, like, “Well, I just don’t want to teach you because you’re being difficult,” and more of like, “I don’t understand how to teach you.”

Another said, “Everyone, teachers hated me. It was like the number one complaint. Like, how do we get [name] to stay in his desk, right? And it’s like, well, how about you just meet [name] in the middle, you know? [laughs].” A third participant compared learning at home online versus the strict rules of the classroom: “I have much freedom in my bedroom than the teachers telling me what to do—I tend to move around my bed stimming and my legs and very relaxing.”

## Discussion

The probe that asked the participants to send a message to their high school aged self resulted in three of the four participants sending messages of comfort through the promise of future success. Schalk (2018), following Siebers, argues that disability studies must show both the positive and negative sides of disability, resisting too much of one by advocating the other. Here, this is played out as the participants advocate for the life events that their autistic nature aids in achieving, or at least does not foreclose on, to resist the negative that they remember experiencing in high school.

The participants also used this probe to give their younger selves hope that their existing strained relationships would improve and/or they would form new, strong bonds. In one case, the participant reassured themself that their sexual identity (asexual, aromantic) was not a flaw, or a sign of brokenness. Minich (2016), in her argument for the methodology of disability studies, notes that it is the social norms of the participant’s high school world that make her non-normative sexual identity an issue for her younger self. Using this methodology, it is possible to scrutinize these “social conditions that concentrate stigmatized attributes in particular populations”—specifically the high concentration of autistic people who have non-normative sexual identities and how that combined identity leads to additional stigma. This may be the source of the text indicating that the participant’s parents do not take their needs seriously.

Participant responses to the shared reading were positive, and they indicated they often felt the protagonist’s actions were relatable. One participant, who did not generally enjoy science fiction, reported, “But, like, this book, I found I’ve enjoyed it a little bit more because I find myself identifying with some things the character says about how they feel in certain situations.” Not all of the feelings raised by the main character’s experiences were positive for the participants. One reported,

And like that’s another thing about the book, at least in the reading that we’ve done so far. Xandria’s mother outright says that people like you don’t actually get to be adults. That was just, ugh. That hit close to home. Like nobody outright said that to me. But it does feel like that a lot of the time. Because I am this way. Even if they don’t know I have the label, they just feel free to disregard and infantilize, blah, blah, blah. I am not to be taken seriously or given proper autonomy.

Schalk (2020), referring to Sara Palmer’s review of the movie *Avatar*, notes that a theme of speculative literature is “compulsory able-bodiedness—that drive toward increased ability at all costs due to our cultural inability to imagine the positive aspects of living with a disability” (p. 139). This novel presents such a setting, but with a main character who (for reasons explained in the novel) was born with a disability (autism). There is a scene where a character (a general) suggests providing their technology to prevent “undesirable” traits in offspring to a newfound race in front of the main character. One participant responded to this by saying “So the general guy, he went right into the eugenics thing and oh boy! . . . He almost just called her a defect right to her face.” The participant also tied the events in the book to the real-world issues of increased accessibility to assisted suicide (MAID) being debated by the federal government. She said,

But like, then you see it on the news just like how many people are being recommended to go on MAID just because like they’re disabled. Their payments aren’t going to be enough to actually live. So they’re just going to like, you know, I can’t deal. I think we are getting very off topic, but like, you know how it is. This is what happens when you talk about a book about eugenics and in space and blah, blah blah.

Following Dokumaci’s (2018) idea of micro-activist affordances, some of the participants described how they prepare the words they are going to speak by practicing them over and over in their head so they come out “correctly” to a non-autistic audience. While the environmental affordances of spoken communication anticipates free flowing conversations, pre-scripting is a way for them to appear to engage in the “proper” way.

The most common means of avoiding conflict that came up was masking—acting in a way that would not draw attention to their differences. Researchers are beginning to study the harms that come from the “suppression of natural responses and adoption of alternatives” (Pearson & Rose, 2021, p. 53).

## Limitations

At the time of writing, the research described here was only partially completed. In addition, the participants were all university students, meaning that they had successfully found ways to meet the requirements of high school and enter post-secondary education. This is not the experience for all autistic high school students.

## Conclusion

Cultural probes, as a means to better understand research participants, have been used in design since Gaver et al.’s (1999) Presence Project. I seek to use this well-documented approach in a new context: understanding the needs of autistic students in mainstream classes to better support their academic success. As the number of autistic students in mainstream classes increases, teachers will need better tools to support their learning. Using cultural probes is one way to better understand those needs and learn directly from autistic people, instead of through those adjacent to them (Nicolaidis et al., 2019; Pellicano et al., 2019).

Using Shared Reading, I was able to draw on my participants’ experiences in the public education system in a way that was less investigator driven than a survey or structured interview. Using participants’ connection to the text, the way they resonated with the characters’ treatment of the autistic protagonist, and how Xandi responded to the world(s) around her I was able to draw on their “autobiographical recollection” (Longden et al., 2015, p. 5). In drawing on the participants’ pasts, we can work together to design a better future for autistic students. Further work with this group of participants will use speculative worldbuilding (Tomin, 2021a) as a way to being to bring these alternative futures into being. By asking autistic former students what their experiences were like, we can get a better understanding of how to improve the system. Or, giving the final word to a participant, “I just [pause] I wish things—I just wish things were easier for me. Easier for people like me.”

## Notes

1. For an explanation of why autistic people prefer identity-first language, see Brown (n.d.) and Gernsbacher (2017).

2. Originally one of the physical products would be photographs, but those have been replaced with digital images taken with the participant’s phone.
